# Advancements in biosurfactant production using agro-industrial waste for industrial and environmental applications

**DOI:** 10.3389/fmicb.2024.1357302

**Published:** 2024-02-05

**Authors:** Thanigaivel Sundaram, Rasiravathanahalli Kaveriyappan Govindarajan, Saranya Vinayagam, Vasumathi Krishnan, Shankar Nagarajan, Ganesh Raja Gnanasekaran, Kwang-Hyun Baek, Suresh Kumar Rajamani Sekar

**Affiliations:** ^1^Department of Biotechnology, Faculty of Science and Humanities, SRM Institute of Science and Technology, Kattankulathur, Tamil Nadu, India; ^2^Department of Biotechnology, Yeungnam University, Gyeongsan, Gyeongbuk, Republic of Korea; ^3^Department of Biosciences, Saveetha School of Engineering, Saveetha Institute of Medical and Technical Sciences, Chennai, Tamil Nadu, India; ^4^Department of Biotechnology, Kalasalingam Academy of Research and Education, Virudhunagar, India; ^5^Department of Biomedical Engineering, School of Engineering and Technology, Dhanalakshmi Srinivasan University, Tiruchirappalli, Tamil Nadu, India; ^6^Instituto de Alta Investigación, Universidad de Tarapacá, Arica, Chile; ^7^College of Natural Sciences, Arba Minch University, SNNPR, Arba Minch, Ethiopia

**Keywords:** biosurfactants, agro waste, green synthesis, waste to value, biomass, environmental application

## Abstract

The adverse effects of waste generation on the environment and public health have raised global concerns. The utilization of waste as a raw material to develop products with enhanced value has opened up novel prospects for promoting environmental sustainability. Biosurfactants obtained from agro-industrial waste are noteworthy due to their sustainability and environmental friendliness. Microorganisms have been employed to generate biosurfactants as secondary metabolites by making use of waste streams. The utilization of garbage as a substrate significantly reduces the expenses associated with the process. Furthermore, apart from reducing waste and offering alternatives to artificial surfactants, they are extensively employed in bioremediation, food processing, agriculture, and various other industrial pursuits. Bioremediation of heavy metals and other metallic pollutants mitigated through the use of bacteria that produce biosurfactants which has been the more recent research area with the aim of improving its quality and environmental safety. Moreover, the production of biosurfactants utilizing agricultural waste as a raw material aligns with the principles of waste minimization, environmental sustainability, and the circular economy. This review primarily focuses on the production process and various types of biosurfactants obtained from waste biomass and feedstocks. The subsequent discourse entails the production of biosurfactants derived from various waste streams, specifically agro-industrial waste.

## 1 Introduction

Biosurfactants are useful microbial amphiphilic compounds that are surface active and biologically effective for a variety of applications or processes. When growing on water-immiscible surfaces, microbes provide an alternative to chemically manufactured conventional surfactants (Master and Markande, 2023). They are also capable of synthesizing them. A wide range of microorganisms, including filamentous fungus, yeast, and bacteria, create amphiphilic compounds known as biosurfactants. In comparison to surfactants made chemically, biosurfactants have a low critical micelle concentration, are highly active in environments with extreme pH, salinity, and temperature, and are very selective (Nikolova and Gutierrez, 2021). Even though chemical surfactants are extensively employed in many different sectors and goods, they present a number of serious issues and difficulties, such as safety, health, and environmental concerns (Schultz and Rosado, 2019). Chemical surfactants are currently linked to a number of issues, such as their effects on the environment, potential hazards to human health and safety, toxicity, limited bioavailability, formation of algal blooms, microorganism resistance to surfactants, and the bioaccumulation of different particles in equipment and pipelines that can cause inefficient performance. Alternative surfactants, such as biosurfactants like natural, biodegradable surfactants and green surfactants (sustainable and environmentally friendly substitutes), are becoming more and more popular as a solution to these issues with chemical surfactants (Jahan et al., 2020). Furthermore, companies are examining innovative and research-based approaches to enhance surfactant compositions and lessen their environmental impact (Johnson et al., 2021).

Industries and regulatory authorities can adopt a number of suggested tactics and solutions to address the issues related to chemical surfactants. These remedies seek to enhance safety, lessen the impact on the environment, and encourage the use of more environmentally friendly surfactants (Badmus et al., 2021). Following are a few important suggestions for remedies: The shift from chemical surfactants to biosurfactants, such as those derived from plants or from microbiological processes, is facilitated by microorganisms. These biodegradable surfactants are less hazardous and have a lower propensity to remain and accumulate in ecosystems (Bolan et al., 2023). Improved biosurfactant compositions and the use of environmentally appropriate packaging for surfactant goods can both decrease plastic waste and increase sustainability in the life cycle evaluation.

**Figure 1:**
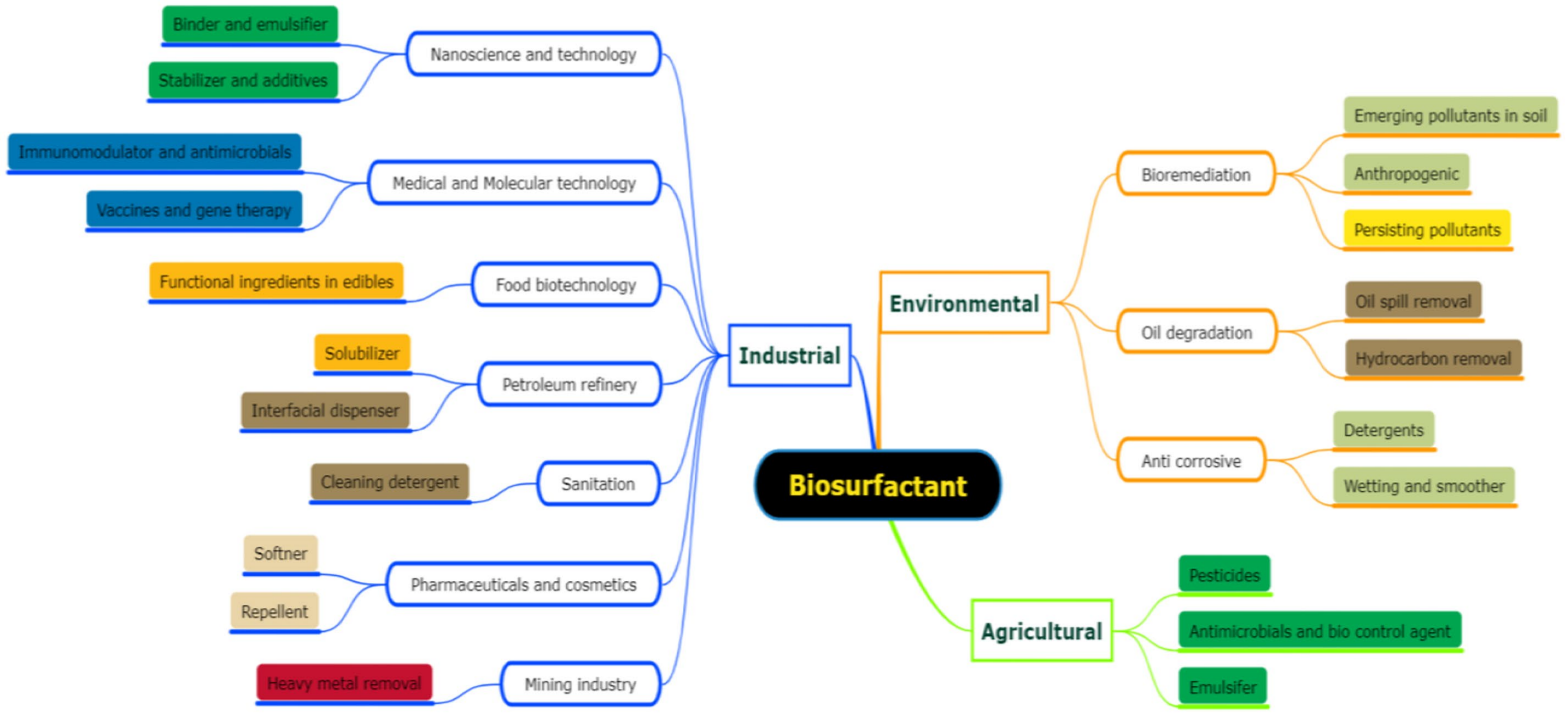
Application of biosurfactant in various field of science and engineering.

Biosurfactants are highly advantageous to society since they are safer than synthetic surfactants, enhance industrial operations, lower pollution levels, and promote progress in a number of industries, including biotechnology, healthcare, and agriculture (Tiwari and Tripathy, 2023). A more ecologically aware and sustainable society could result from their widespread adoption. Because of their low toxicity and biodegradability, these compounds have a wide range of applications in the pharmaceutical, alimentary, and cosmetic industries as humectants, emulsifiers, preservation agents, and detergents as detailed types were illustrated in the Figure 1.

When biosurfactants are used instead of synthetic surfactants, which are harmful to the environment, they have several advantages for the environment, including biodegradability and an environmentally benign approach (Gayathiri et al., 2022). They also reduce toxicity and can be used later in environmentally sensitive locations. Aside from their well-known bioremediation approach, which improves the solubility and bioavailability of hydrophobic contaminants, they demonstrate greater oil recovery in the oil and gas business with improved efficiency from diverse reservoirs (Karnwal et al., 2023). These biosurfactants are essential to the constructive pattern in a variety of biotechnological applications, including the food industry, pharmaceutical, cosmetic, healthcare, and medical drug delivery, as well as agricultural pesticide formulations (Wang et al., 2014). Various substrates can be used to make them, with the majority of the materials being renewable resources such vegetable oils, distillery waste, and milk waste. The developments and recent breakthroughs in the utilization of different types of industrial agriculture waste as a renewable raw substrate material for the synthesis of biosurfactants and novel uses for them (Abdel-Mawgoud et al., 2010). The several wastes and byproducts from agro-industrial processes that are utilized to produce biosurfactants are also included in this review. These include wastes from fruit and vegetable processing, oil processing wastes, starch wastes, sugar industry wastes, distillery wastes, and much more.

### 1.1 Biosurfactant: types, classifications

Organic substances that are surface-active and generated by plants, animals, or microorganisms are typical biosurfactants. There are several varieties of them, and each is categorized based on unique chemical structures, secondary metabolite form microbial origins, and characteristics. Based on the following criteria, these kinds and classifications are formed (Sałek and Euston, 2019; Rusu et al., 2023). Rhamnolipids, sophorolipids, and trehalolipids are the best examples of glycolipids, lipopeptides, phospholipids, polymeric biosurfactants, protein, and peptides, various other types of biosurfactant produced form the agro waste and derived products have been listed in the Table 1. They have been classified as glycolipids based on their chemical structure, which is embedded with the hydrophilic head group linked with hydrophobic fatty acid tail. Bacterial, fungal, and other biosurfactants that are created by bacteria, yeast, and other microorganisms, respectively, are categorized according to the microbial origin and the type of microbe that creates them (Adetunji and Olaniran, 2021). Additionally, biosurfactants can be classified as low molecular weight surfactants, which lower surface tension and improve emulsification; bio emulsifiers, which encourage the formation and stabilization of emulsions; foam-forming biosurfactants, which produce stable foams of biosurfactants; and antimicrobial, which usually strengthen the antimicrobial properties against pathogens (Thakur et al., 2020). This classification is based on how biosurfactants operate in various systems.

**Table 1:** Types of biosurfactant produced form the agro waste and derived products.

Type	Microorganisms involved	Properties	Applications	References
Rhamnolipids	*Pseudomonas aeruginosa*	Emulsifier	Bioremediation, Oil recovery	Neto et al. (2009)
Sophorolipids	*Candida bombicola*	Emulsifier	Detergent, Personal care products	Jiménez-Peñalver et al. (2019)
Trehalolipids	*Rhodococcus species*	Wetting agent, Emulsifier	Bioremediation, Agriculture,	Thakur et al. (2020)
Saponins	*Aspergillus, Bacillus*, etc	Detergents	Improve soil nutrient availability	Góral and Wojciechowski (2020)
Lipopeptides	*Bacillus* species	Antimicrobial	Pharmaceutical and cosmetic industries.	Zhang et al. (2022)
Exopolysaccharides	Lactic acid bacteria	Texture	Thickeners, stabilizers in food industry	Leong et al. (2021)
Bioemulsifiers	*E. coli, Acinetobacter junni, Aeromonas caviae, Pseudomonas fluorescens, Klebsiella pneumonia, Bacillus sp*	Emulsifier	Improve food stability and shelf life	Sałek and Euston (2019)
Polymeric Biosurfactants	*Pseudomonas* spp. and *Bacillus spp*	Emulsan of polymeric substances	Bioremediation and food industry	de Oliveira Schmidt et al. (2021)
Lipopeptides	*Brevibacillus brevis, Bacillus* sp.	Antipathogenic	Bioremediation and medical	Sarubbo et al. (2022)
Surfactin	*Endophytic Bacillus* sp.	Antifungal	Bioremediation and medical	Vieira et al. (2021)

## 2 Biosurfactant properties and production methods

The molecular structures and activities of biosurfactants vary widely. Hydrocarbon chains of various lengths and complexity make up the nonpolar hydrophobic tail, whereas peptides, carbohydrates, amino acids, alcohol, or phosphate carboxyl acid make up the polar hydrophilic head (Asgher et al., 2020). It is made from surfactants derived from biological entities, especially bacteria and fungi, and can be made in a number of ways, the main ones being with microorganisms or plant-based sources. These substances are produced either as metabolic byproducts or as a result of the chemistry on the cell surface (de Oliveira Schmidt et al., 2021). These substances have been shown to provide a preferential partition between liquid interfaces with different polarities, such as oil/water/air, and to improve the bioavailability of substrates by lowering surface and interfacial tension. Additional physiological characteristics are a wide range of structural variations, minimal toxicity, compatibility with the environment, increased biodegradability, stability at high and low temperatures, salinity, and pH, low critical micelle concentration, and a wider range of substrate specificity (Kumar et al., 2022). Several synthetic surfactants are surpassed in their ability to moisten, detergency, micro-emulsify, foam, separate phases, and exhibit selective tension-active properties when compared to microbial biosurfactants. The choice of microbe or source, the desired application, and the specific type of biosurfactant all have an impact on the manufacturing method of biosurfactant (Kumar et al., 2022). The following section has covered a few popular techniques for producing biosurfactants. Biosurfactants are among the most flexible bioproducts of contemporary biotechnology because they can be produced from microbial sources in both constitutive and inducible forms. Moreover, it is possible to improve the process economics of biosurfactants for bulk the production by using renewable and inexpensive raw materials, industrial wastes, or other byproducts.

### 2.1 Microbial fermentation

**Figure 2:**
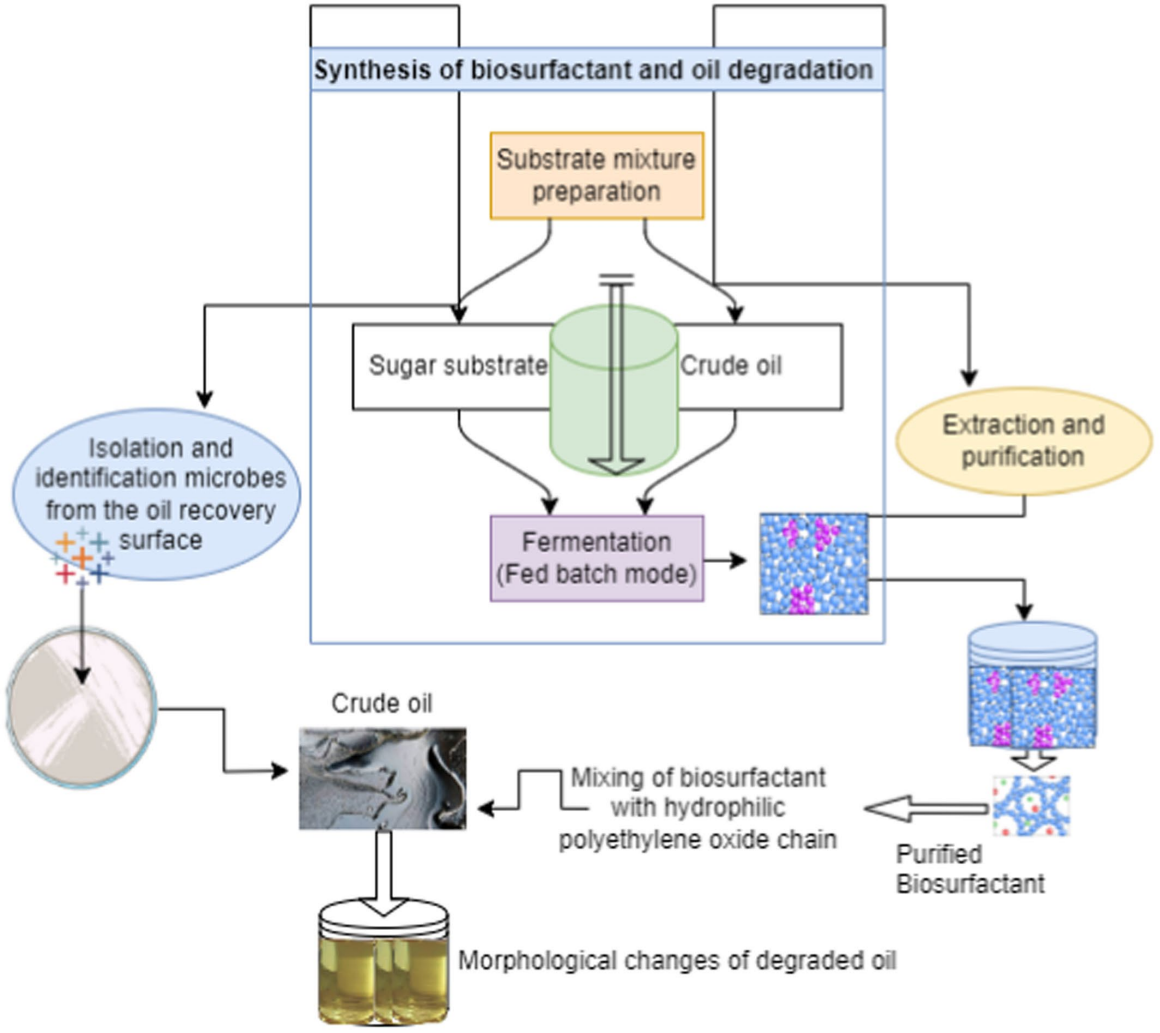
Biosynthesis of surfactant and its application in oil degradation.

Biosurfactants are widely distributed because they are formed by bacterial and eukaryotic species. They are surface-active chemicals that are either secreted extracellularly or generated at the surfaces of live cells. Numerous microbial, animal, and plant species have the ability to create biosurfactants (Zahoor et al., 2022). The use of biosurfactants allows microbes to more easily get hydrocarbons and other insoluble substrates into their cells. Lecithins, saponins, and soy proteins are a few plant-based biosurfactants that have shown great emulsification capabilities. However, their high cost and additional problems with hydrophobicity and solubility make their large-scale manufacturing impractical. The most popular technique for producing biosurfactants is this one (Thakur et al., 2020). It entails growing microorganisms in an appropriate growth medium, like yeasts or bacteria. The bacteria create biosurfactants as metabolic byproducts during fermentation. Temperature, pH, and oxygen concentrations during fermentation are all optimized to produce biosurfactants. Microbes that frequently produce biosurfactants include *Bacillus*, *Candida*, and *Pseudomonas* (Schultz and Rosado, 2019). This type of microbial fermentation uses a variety of process techniques, including batch and continuous fermentation (López-Gómez et al., 2019). Microorganisms are grown in a closed environment with a set volume of growth medium in batch fermentation (Olszewska-Widdrat et al., 2020). Inoculation, growth, and the synthesis of biosurfactants are the steps in the process, the detailed scheme of the synthesis has been presented in the Figure 2. Harvesting and downstream processing used to maintain a steady-state culture during continuous fermentation, fresh growth media must be added on a regular basis and culture broth must be removed (Márquez-Villa et al., 2022; Xu et al., 2023). In addition to these techniques solid state fermentation, submerged, plant-mediated, and, more recently, genetic engineering-based techniques it is frequently employed to increase biosurfactant production and productivity (Sodhi et al., 2022). This technique involves the cultivation of microorganisms on solid substrates like oilseed cakes or agricultural leftovers. For some fungal biosurfactants in particular, solid-state fermentation is an excellent option. Microorganisms develop and generate biosurfactants in a liquid media during submerged fermentation (Kim et al., 2023). It is extensively employed in the synthesis of yeast and bacterial biosurfactants. Plant sources can yield certain biosurfactants, like saponins. Plants with a high saponin content, such as quillaja trees and soapnuts, can be used to produce biosurfactants (Mondal et al., 2023). Microorganisms can produce more biosurfactants by using genetic engineering approaches. It is possible to change the characteristics of biosurfactants and boost yield by modifying the genes involved in their synthesis (Góral and Wojciechowski, 2020).

#### 2.1.1 Downstream processing of biosurfactant by microorganism

Particularly for biotechnological products with high yields in the upstream synthesis processes, downstream processing is crucial in determining manufacturing costs. Comprising microorganisms in fermentation processes, biosurfactants are surface-active chemicals. To increase the synthesis of rhamnolipids upstream, numerous approaches in fermentation engineering, process design, and recombinant technologies have been studied. A low concentration of product in the broth, unknown or insufficient thermodynamic data of various broth and product components, and complexity of numerous component broths and products are the main obstacles encountered in the downstream purification of biosurfactants (Sharma, 2021). The common downstream processing method was tracked by tracking the quantity of rhamnolipids as they went through the unit operation steps in the study by Invally et al. (2019). However, the presence of residual oily substrates and metabolites in the fermentation broth caused complications, which were taken into consideration and their effects on the downstream process were presented. The total economic viability of rhamnolipid production as an industrial product is enhanced by advancements in downstream processing (Neto et al., 2009). As a prime example, Rhamnolipids were examined for the downstream production process due to their significance as biosurfactants and their numerous, potentially advantageous industrial and environmental uses. Significant effects were observed on the necessary downstream purification procedures for rhamnolipids due to the remaining oil in the harvested fermentation broth. However the downstream purification of is still not obvious in many of the investigations (Invally et al., 2019).

### 2.2 Co-production method with other products

In biorefinery processes, biosurfactants can be co-produced alongside other useful products as biofuels or enzymes. This method makes the best use of the resources (Gallego-García et al., 2023). These techniques make use of bioreactors, like airlift reactors and stirred-tank reactors, which are frequently utilized for the large-scale manufacture of biosurfactants. They offer scalability and control over the surroundings. Following the creation of biosurfactant, these processes can be made using the downstream process and the processing parameters. The downstream processing procedures are used to separate and purify the biosurfactant from the culture broth (Dey et al., 2022). In order to increase biosurfactant production yields and lower production costs, processes such as extraction, centrifugation, filtration, and chromatography may be employed (Luft et al., 2020). Optimization approaches, such as statistical experiment design, may then be applied. The choice of production method is contingent upon various aspects, including the particular biosurfactant of interest, the microbe employed, the size of production, and the needs of the application (Vigneshwaran et al., 2021). Each method has pros and cons. The cost-effectiveness, environmental impact, and purity of a biosurfactant should all be taken into account when choosing a production technique (Venkataraman et al., 2021).

### 2.3 Process parameters involved in microbial fermentation

The rapid generation, multifunctional properties, accessibility, and potential for scaling up of microbial biosurfactants make them superior to other biological surfactants. Even while research on microbial biosurfactants began many years ago, in the last few years, these molecules have seen an increase in industrial use (Ahmadi-Ashtiani et al., 2020). Many different types of microbes, including bacteria, fungus, and yeasts, have been engineered to make biosurfactants (Venkataraman et al., 2021). The most important thing to keep in mind is that throughout the growth of the microbial culture, a variety of extrinsic and intrinsic dynamics, the kind of substrate, media supplements, and the microbial species all affect the quantity and quality of biosurfactants (Invally et al., 2019). A popular technique for producing biosurfactants as metabolic byproducts is microbial fermentation, which includes growing microorganisms in a regulated environment. The entire production process from choosing the microbe to characterizing it and scaling up the final product is covered (Gallego-García et al., 2023). To prepare the inoculum, a small amount of a well-characterized stock culture is transferred from a stock culture to a fresh growth medium. The process starts with the selection of common microbial strains, such as *Pseudomonas* spp., *Bacillus* spp., *Candida* spp., and others, depending on the type of biosurfactant desired (Phulpoto et al., 2020). Following the appropriate monitoring and control procedure, the inoculum is allowed to develop and adapt to the medium, and then the fermentation medium is prepared and the biosurfactant is extracted (Jimoh and Lin, 2019). If larger fermentation tanks or bioreactors are needed, the production process can be scaled up for industrial uses. It is noteworthy that the particular parameters and conditions pertaining to microbial fermentation will differ based on the kind of microbe and biosurfactant being generated (Márquez-Villa et al., 2022). Achieving high yields and high-quality products requires the optimization of these characteristics. Furthermore, during the production process, compliance with safety and legal requirements is crucial (Nikolova and Gutierrez, 2021).

## 3 Genetic engineering in biosurfactant production

**Figure 3:**
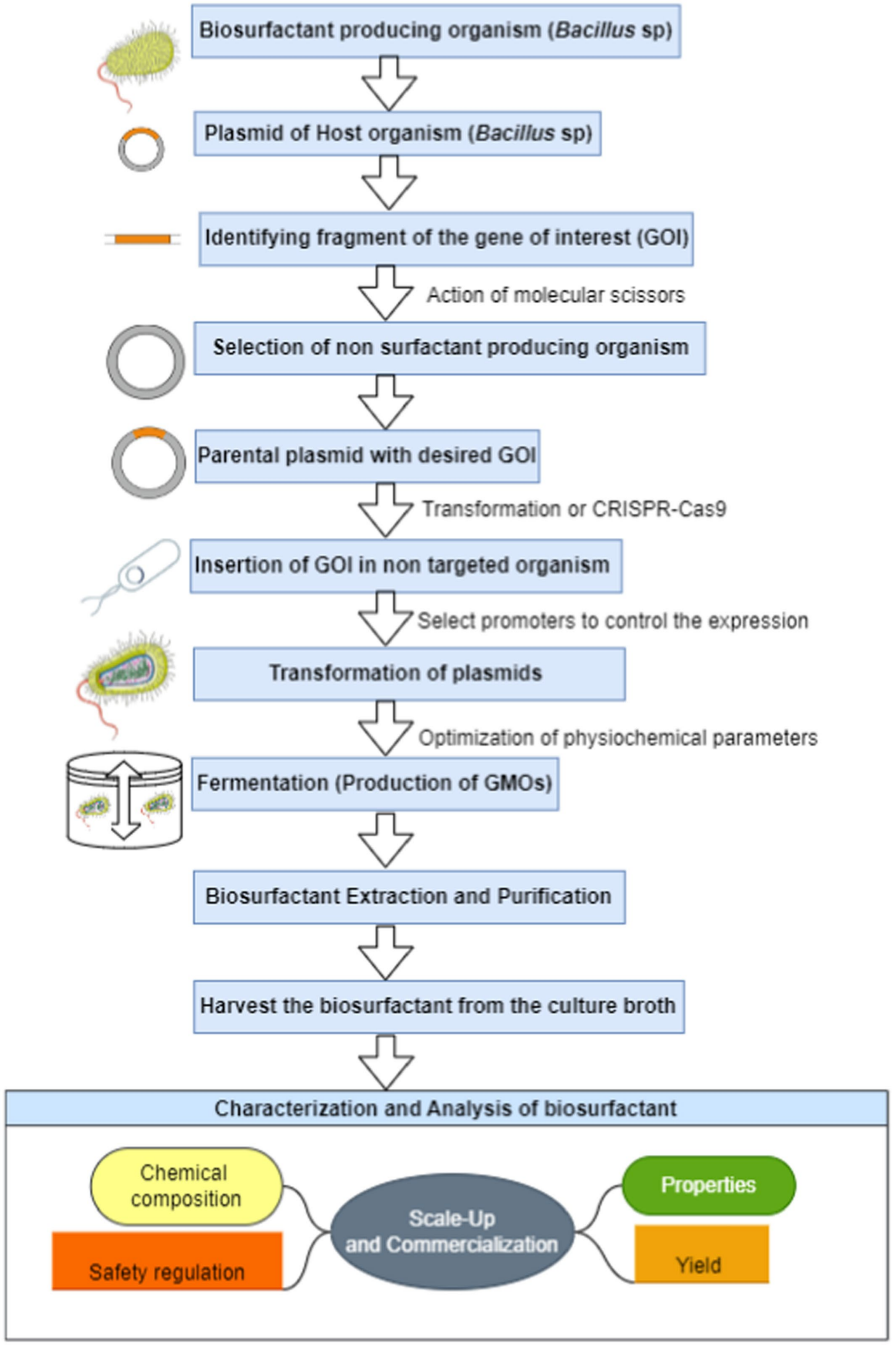
Molecular steps involved in the biosurfactant production by genetic engineering.

Genetic engineering (GE) produces biosurfactants by modifying microorganisms to increase their capacity to produce biosurfactants. Biosurfactant yield, characteristics, and production efficiency can all be optimized with this technique (Jimoh and Lin, 2019). Yeast, fungus, and bacteria are frequently used in the GE method’s biosurfactant production process, which is comparable to the microbial fermentation process (Thakur et al., 2020). The genetic resources available for the selected microbe and the intended biosurfactant will determine the selection. The process of discovering and isolating the genes necessary for biosurfactant production from naturally existing bacteria that produce biosurfactants is a critical component of biosurfactant research (Schultz and Rosado, 2019). Enzymes and proteins involved in the creation of biosurfactants are usually encoded by these genes. Molecular procedures will introduce the biosurfactant genes into these encoded genes, changing their genome (Jimoh et al., 2021). Transformation, transfection, and CRISPR-Cas9 gene editing are a few genetic engineering methods that can be used to accomplish this (Mgbechidinma et al., 2022; Bokade et al., 2023). After the promoteo region is expressed, insert the biosurfactant genes into the host microorganism’s chromosome or plasmids, which are small, circular DNA molecules that can replicate independently. This will allow for the development of genetically engineered strains that can be released for commercial use by standardizing and enhancing the microorganism’s growth (Gaur et al., 2022). Choosing the proper growth medium, temperature, pH, and aeration are all part of this process. The benefit of genetic engineering is its capacity to modify biosurfactants to suit particular industrial and environmental requirements, the detailed steps involved in the role of genetic engineering in the biosurfactant production is presented in the Figure 3. This can be used to improve their performance, lower manufacturing costs, and address sustainability issues. To guarantee appropriate and moral use, it also necessitates a deep comprehension of genetic processes and safety concerns (Mgbechidinma et al., 2022; Krucoń et al., 2023).

### 3.1 Variable impacts affect the biosurfactant production

Numerous factors can impact the yield, content, and qualities of the biosurfactants produced during the production process. These variables can change based on the particular microbe, the environment in which it grows, and the kind of biosurfactant that is being generated (Habib et al., 2020). These determining elements are frequently the kind of bacterium, since distinct strains differ in their capacity to produce biosurfactants. The availability and mix of nutrients in a growing medium have a major influence on the generation of biosurfactants, and strains are chosen according to their genetic make-up, productivity in producing biosurfactants, and suitability for the intended use (De Giani et al., 2021). In particular, supplies of carbon and nitrogen are necessary for the growth of microorganisms and the synthesis of biosurfactants. The concentration of carbon sources, such as sugars, hydrocarbons, or organic acids, can also affect the synthesis of biosurfactants (Sánchez, 2022). For the best biosurfactant production, some microbes favor particular carbon sources. Whether the nitrogen source is organic (like peptone or yeast extract) or inorganic (like ammonium salts), it has a significant impact on the synthesis of biosurfactants. Sources of nitrogen can affect metabolism and cell growth. Another essential element for microbial development and the synthesis of biosurfactants is the growth medium’s pH level (Kumar et al., 2022). There are pH ranges where certain microbes can synthesize biosurfactants well. The rate of growth and metabolic activity of bacteria are influenced by temperature. Several strains have various ideal temperatures for the synthesis of biosurfactants. Aeration and oxygen, however, are important for microbial metabolism, and the amount of oxygen present in the fermentation vessel might influence the generation of biosurfactants. Various biosurfactant profiles can result from anaerobic and aerobic environments (Kashif et al., 2022). Cell development and the formation of biosurfactants are influenced by the agitation and aeration rates in bioreactors or fermentation containers. Optimizing the yields of biosurfactants requires careful mixing and oxygen delivery (Sodhi et al., 2022). Numerous additional factors, including salinity, the ratio of carbon to nitrogen, the size of the inoculum, genetic alterations, substrate concentrations, and different environmental stressors, were impeding the process of production (Gayathiri et al., 2022). For the manufacturing of biosurfactants to be done efficiently, it is imperative to comprehend and optimize each of these factors. To attain the intended biosurfactant yield and qualities, a mix of these variables may need to be adjusted, depending on the particular biosurfactant and microorganism (Zhang et al., 2022).

### 3.2 Challenges in the biosurfactant production

Production of biosurfactants is confronted with a number of obstacles that could prevent their general acceptance and commercialization, despite their potential for a variety of industrial and environmental uses (Saraç et al., 2022). The following are some of the major obstacles in the manufacture of biosurfactants. The primary barrier to the production of biosurfactants is feedstock selection (Jahan et al., 2020). Biosurfactant formation is influenced by composition in addition to higher substrate concentrations. The nitrogen concentration, protein sources, fatty acid content, glucose, sucrose, and other components of industrial waste can be used to establish the feedstock priority.

Furthermore, it affects the amount of biosurfactant that is retrieved in the next phase. Industrial waste selection compromises the media content. There should be minimal pre-treatment and purification required of industrial waste feedstocks to decrease operating and capital expenses and the generation of obstacles like acetic acid (Santos et al., 2023). Industrial waste primarily has three problems: pre-treatment, transportation costs, and raw material accessibility. Industrial waste contains unwanted components; thus, it requires additional procedures in waste treatment, the most important of which are clarification before and after fermentation. Once such pretreatment is completed, the feedstocks ought to have substrates for straightforward formulation. As production costs are being reduced, reduced pre-treatment should also be considered. Industrial wastes like sugar effluent and oil waste are examples of such feedstocks (Wang et al., 2014). Economic factors need to be considered in order to raise the likelihood of commercial viability. The previously specified substrates should be available in sufficient quantities and at a fair price in the locality for the pricing to remain constant throughout the year. In the process of producing biosurfactants, storage and transportation are significant financial considerations. Newer studies should focus on increasing the number of beneficial bacteria in order to increase the production of biosurfactants from less-treated industrial waste. Future waste treatment and product production should be accomplished through the use of recombinant microorganisms (Ciurko et al., 2022).

## 4 Types of sources used for biosurfactant production

Based on the techno-economic feasibility, biosurfactants cannot compete with synthetic chemicals, three drawbacks were faced by them: production, functioning, and cost. Unfortunately, biosurfactants cannot be used in a wide range of applications due to their high production costs (Jahan et al., 2020). Therefore, different approaches need to be developed to lower production costs. For the growth of organisms and the manufacture of biosurfactants, cost-effective bioengineering techniques and renewable substrates must be used. The carbon and nitrogen sources, which lower manufacturing costs, are the most significant components of biosurfactant production. The primary constraints on the manufacture of surfactants are their decreased output and increased manufacturing costs, which limit their use in a variety of applications (Jahan et al., 2020).

### 4.1 Agricultural waste

**Figure 4:**
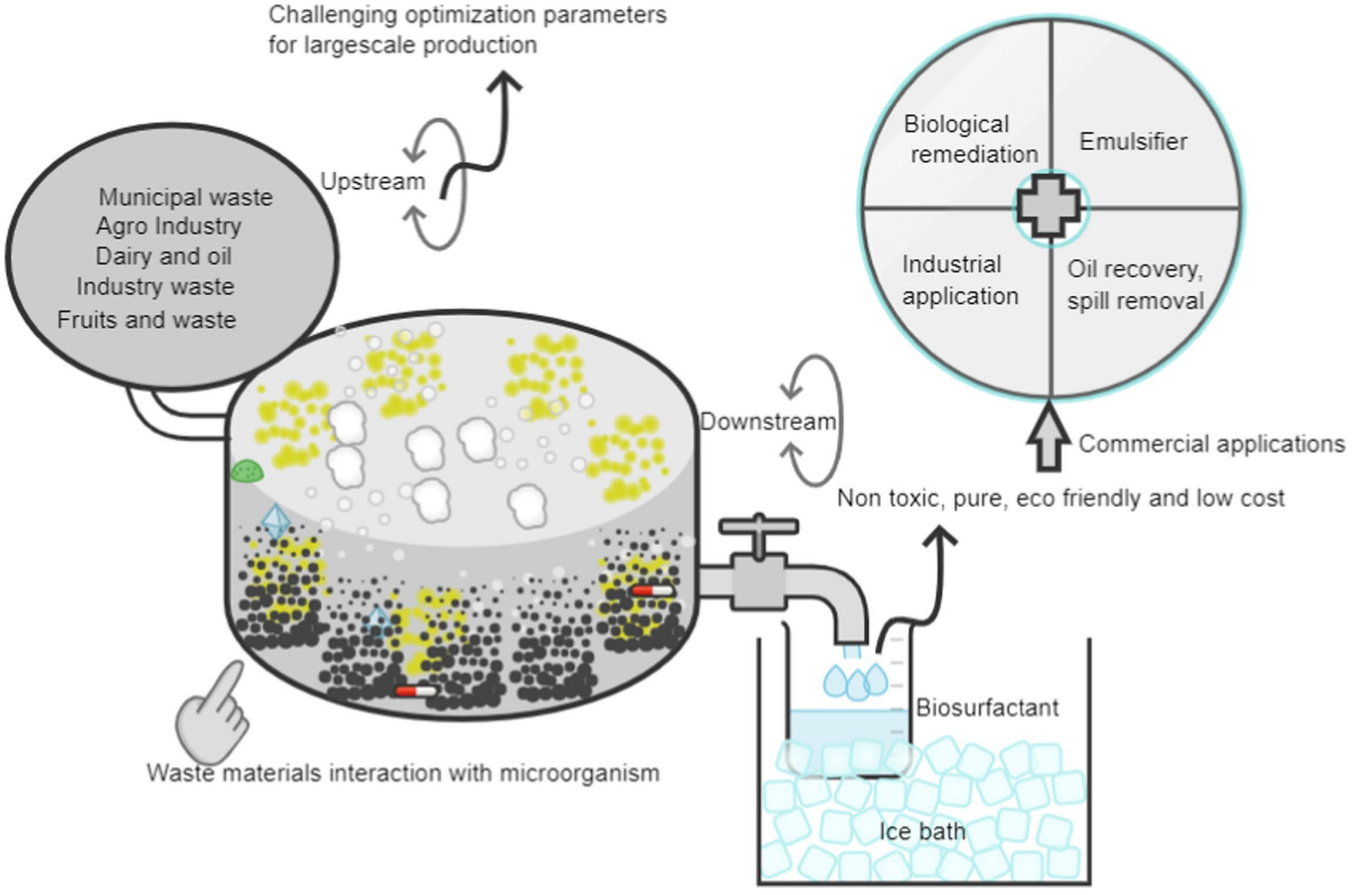
Biosynthesis process of biosurfactants using waste different wastes.

Using agricultural waste to produce useful biosurfactants is a sustainable and eco-friendly way to make use of organic wastes. The general stages in producing biosurfactants from agricultural waste are outlined here (Santos et al., 2023). This approach uses agricultural waste materials as the substrate for the synthesis of biosurfactants, the schematic illustration of the biosynthetic strategies is shown in the Figure 4. Crop residues (such as rice husks and wheat straw), fruit and vegetable peels and agro-industrial outputs (such as sugarcane bagasse and maize cobs) are examples of common agricultural wastes (Ciurko et al., 2022). In addition to adding value to organic leftovers, the manufacture of biosurfactants from agricultural waste encourages sustainable practises and lessens the environmental impact of trash disposal. In order to successfully produce biosurfactants from these renewable resources, process optimization and the selection of agricultural waste and microorganisms will be crucial (da Silva et al., 2021).

### 4.2 Fruit and sugar industry waste

Peels, pulps, and seeds are among the many organic waste products produced by the fruit companies. If this trash is not managed well, it may cause problems for the environment. Nonetheless, creative methods have surfaced to transform this waste into useful goods, such as the synthesis of biosurfactants (Helvia et al., 2017). Fruit peels, pulps, and seeds are among the many waste products produced by the fruit processing industry. These materials are frequently thrown away or handled as agricultural waste. These residues are good substrates for the synthesis of biosurfactants because they are high in proteins, lipids, and carbohydrates. Fruit waste can be used to produce biosurfactants, which not only solves trash disposal issues but also adds value to a resource that would otherwise go unused (Sharma and Pandey, 2020). The generation of biosurfactants from fruit industry waste is an environmentally responsible and sustainable method of managing waste and using resources. In addition to easing garbage disposal issues, it has several positive economic and environmental effects. Using this green technology promotes a more ethical and ecologically aware fruit sector by being in line with the concepts of sustainability and the circular economy. Further research and development in this area should lead to the widespread use of biosurfactants made from fruit waste in a variety of industrial applications in the near future (Sharma and Pandey, 2020).

Wastewater containing starch is produced in enormous quantities by the sugar processing industries. In addition to sugarcane, a variety of other resources are utilized in the commercial manufacture of sugar, including tapioca, corn, soybean, potato peel powder and wheat bagasse. The massive volumes of glucose and fructose found in the sucrose-rich sugarcane molasses are immense (Banat et al., 2000). The kind of crop and the processing techniques used determine the variances in the nutritional makeup of the molasses. Using molasses lowers the costs associated with pre-treatment, water use, and logistics. With the exception of solid removal and dilution, they are simple to use in the manufacturing of biosurfactants (Vieira et al., 2021). The possibility of making biosurfactant from sugar byproducts has been the focus of some studies. Because of its high vitamin content, molasses is the substrate that is employed most frequently. Several recent reviews have examined different aspects of biosurfactant types and their role in a wide range of bioremediation applications, including dye degradation, hydrocarbon breakdown, heavy metal extraction, and oil removal (Carolin et al., 2023). The biosurfactants derived from molasses feedstock exhibit superior surface tension and emulsification capabilities, indicating their potential for use in bioremediation projects (Begum et al., 2023). Moreover, it improves the ability of microorganisms to break down and decompose various persistent pollutants found in different environments, such as soil and water (Saisa-Ard et al., 2013).

### 4.3 Wheat straw and rice husks as feedstocks

An environmentally safe and sustainable method of producing valuable biosurfactants from biomass waste is the synthesis of biosurfactants from agricultural leftovers such as rice husks and wheat straw (Rodríguez et al., 2021). In the process of making cheese and casein, liquid material known as whey is produced. With its high protein and carbohydrate content and trace amount of fat, whey is a highly nutritious food. Whey is basically made from two different types of dairy products that are sold commercially. Biodegradable surfactants with a range of uses are also produced by this method, which lessens the environmental impact of agricultural waste (Domínguez Rivera et al., 2019). Rich in nutrients and frequently seen as waste materials, agricultural leftovers include rice husks and wheat straw. Burning them or letting them break down causes problems with disposal and pollution. Nonetheless, the complex carbohydrates found in these lignocellulosic materials cellulose, hemicellulose, and lignin can be processed to produce useful byproducts like biosurfactants (Rajasimman et al., 2021).

A number of obstacles stand in the way of the promising potential for producing biosurfactants from rice husks and wheat straw. Increasing biosurfactant production, maximizing fermentation efficiency, and guaranteeing cost-effectiveness are a few of these (Helvia et al., 2017). Further work is being done to better understand genetic engineering methods and find novel microbial strains for increased biosurfactant production (Gong et al., 2021). A practical and sustainable method for turning agricultural waste into valuable products is to produce biosurfactants from rice husks and wheat straw. In addition to addressing waste disposal issues, this biotechnological technique supports green chemistry projects by providing environmentally friendly surfactant substitutes for synthetic ones. Making biosurfactants from agricultural leftovers has significant potential to contribute to the development of a more sustainable and circular economy as long as research and technology continue to progress (Sałek and Euston, 2019).

### 4.4 Dairy waste

The presence of a significant amount of organic matter in dairy waste presents a substantial environmental hazard. If not handled correctly, it can lead to pollution and the release of greenhouse gases. On the other hand, the idea of using dairy waste to make biosurfactants has recently gained a lot of attention (Leong et al., 2021). The volume of dairy waste generated by activities involving the use of dairy products is substantial. Dairy wastewater is better suited to the kinds of operations carried out in the industry due to its composition and characteristics. The characteristics of wastewater from dairy factories vary significantly in this aspect. The term “dairy waste” encompasses a range of residual materials generated during the processing of milk, cheese, and yoghurt (Sar et al., 2022). The inclusion of lipids, proteins, lactose, and other organic compounds in these by-products renders them ideal substrates for bacteria that generate biosurfactants (Jiménez-Peñalver et al., 2019). The primary constituents of dairy waste that are utilized in the production of biosurfactants are proteins and lipids derived from milk. Dairy effluent’s lactose and protein promote microbial development, which in turn leads to the generation of valuable byproducts (Leong et al., 2021). Utilizing dairy waste for the production of biosurfactants is an ecologically conscientious and enduring approach to waste management. It has diverse applications in other industries, apart from its ability to transform dairy waste into a profitable resource. The production of biosurfactants from dairy waste is an innovative and stimulating approach that promotes waste minimization and the adoption of eco-friendly methodologies, in response to the global demand for ecologically sustainable solutions to address ecological issues (Hajam et al., 2023).

### 4.5 Applications in oil industry

Oil byproducts include a variety of substances, including lipids, oil seed cakes, fatty acid residues, water-soluble effluents, and soap stocks. The oil processing business generates a wide variety of waste products, such as tallow oil, free fatty acids, soap stock, and marine oils. Researchers are particularly interested in this topic because the release of such wastes raises more concerns (Varjani et al., 2021). Because they can modify interfacial characteristics, improve emulsification, and lower surface tension, biosurfactants are useful in a variety of oil industry applications. The oil industry uses biosurfactants for a number of important purposes, such as enhanced oil recovery (EOR), biodegradable and environmentally friendly, oil spill cleanup, oil desorption from solid surfaces, and Microbial Enhanced Oil Recovery (MEOR) (Liu et al., 2021). Accordingly, biosurfactants are used to increase the recovery of oil from reservoirs and are an essential part of the enhanced oil recovery process (Du et al., 2020). They facilitate the flow of oil through porous rock formations and its displacement by injected water or gas by lowering the interfacial tension between oil and water (Nasiri and Biria, 2020). Oil fields can have their lifespan extended and oil production increased by this procedure. If there is an oil spill in a marine setting, biosurfactants can be used to emulsify and spread the oil, lessening its effects on the ecology (Adetunji et al., 2023). They facilitate the development of oil-in-water emulsions, which facilitates the containment and removal of the oil. Because biosurfactants lessen the adherence of oil to surfaces, they can be used to desorb and remove remaining oil from solid surfaces, such as equipment or rocks polluted with oil. However, biosurfactant-producing microbes are injected into oil reservoirs as part of MEOR procedures in order to manufacture biosurfactants on-site (El-Sheshtawy et al., 2015). These biosurfactants then modify wettability and reduce interfacial tension, which aid in the mobilization and recovery of trapped oil. In the oil sector, biosurfactants are frequently chosen over synthetic surfactants due to their environmental effect and biodegradability. This is consistent with the industry’s endeavors to embrace ecologically sustainable methodologies. Biosurfactants play a major role in the oil industry’s utilization of microorganisms and their metabolic activities to enhance oil recovery from reservoirs, as well as in the bioremediation of oil contaminations (MEOR) (Aboelkhair et al., 2022). Utilizing microorganisms and their metabolic processes, bioremediation of oil contaminations is a technique used to break down or purify environmental pollutants, such as hydrocarbon and oil contaminants. Enhancing the efficiency of bioremediation is largely dependent on biosurfactants. Achieving excellent results in bioremediation and MEOR applications depends on the selection of suitable microorganisms and biosurfactants, as well as the optimization of environmental conditions (Accorsini et al., 2012).

## 5 Future prospects

Biosurfactants, despite their numerous applications across different industries, suffer from low productivity and high expenses, posing challenges for their extensive commercial utilization (Thakur et al., 2020). In order to propagate colonies and generate products, microorganisms generally require water, vitamins, minerals, carbon and nitrogen sources, as well as ideal physicochemical conditions. By using essential components necessary for the growth of microorganisms, it is possible to readily produce a culture medium using small-scale techniques (Kumari et al., 2023). However, it is not possible to get an equivalent level of success when employing this medium for large-scale production. Here, the culture medium needs to be more selective in its production of the target goods, not the unwanted ones. The ideal incubation medium would not only be easily sterilizable, but also provide a value product with a long shelf life, be able to adapt to various growing settings, and not produce any toxic waste (Silva et al., 2023). The future advancements in biosurfactants show great potential and encompass several significant domains. It is desirable to utilize industrial, agricultural, and food waste, together with outputs from other operations, as substrates in the production processes (Kumar and Karmakar, 2023). The type of biosurfactants formed is determined by both the nutritional and environmental conditions provided to the microorganisms during their growth, in addition to their influence on the biosynthesis process (Bose et al., 2023). Biosurfactants are necessary to enhance the solubility and bioavailability of hydrophobic pollutants. Microorganisms are expected to play a crucial role in bioremediation projects, the cleanup of polluted sites, and the management of water and soil contamination. Biosurfactants are currently attracting significant attention in the pharmaceutical, healthcare, increased oil recovery, and oil spill response industries because to their potential to enhance the solubility and bioavailability of drugs with low water solubility. Recently, the utilization of biofuel and bioenergy has enhanced the efficiency of biotechnological procedures, such as the production of enzymes and biofuel (Raud et al., 2019; Markande et al., 2021). They could contribute to the development of environmentally-friendly bioenergy sources. New uses and benefits for society include the utilization of biopesticides, advancements in space exploration, and improvements in wastewater treatment. The utilization of these waste streams for biosurfactant production leads to enhanced resource efficiency and reduced costs associated with waste disposal (Tang et al., 2023). To mitigate the adverse environmental impacts of the food processing and agricultural industries, as well as address waste management and pollution concerns, we can employ the practise of repurposing waste materials into valuable commodities such as biosurfactants. The circular economy’s fundamental principles, which involve transforming waste into valuable resources and promoting a more sustainable and regenerative economic model, are reinforced through agro based industrial waste for the production of biosurfactants (Venkataraman et al., 2023). The use of agro-industrial waste can enhance local and regional economies by promoting the growth of new value-added firms, generating employment opportunities, and increasing the competitiveness of agro-industrial sectors (Ceresa et al., 2023). Conversely, with the growing significance of environmental sustainability and safety to regulatory authorities, biosurfactants are expected to experience a rise in market expansion and acceptance (Jimoh et al., 2021). Overall, the prospects for biosurfactants are promising, as they are increasingly being utilized in various industries and there is a growing emphasis on environmentally benign and sustainable alternatives. Further investment in biosurfactant technology is expected to enhance its effectiveness and economic feasibility through ongoing research, development, and funding (Coscia et al., 2023).

## 6 Conclusion

A reasonable solution to the environmental and economic problems at hand is provided by using agro-industrial waste as a commercially viable source of raw materials for biosurfactants. This method looks forward to developing more environmentally friendly and sustainable surfactants that have a wide range of uses in various industries. It is in line with the concepts of sustainability and the circular economy. Agro-industrial waste can be fully utilized as a valuable resource for the manufacturing of biosurfactants with more research and development in this area. Biosurfactants has distinctive attributes and eco-friendly qualities, rendering them exceptionally beneficial for a diverse array of industrial and environmental applications. Biosurfactants are known to be more expensive than their chemical substitutes. The application of biosurfactants in the commercial sector is greatly influenced by various elements including substrates, microorganisms, downstream processing, and economic incentives. New opportunities and uses for agro-industrial waste as a source of biosurfactants are being made possible by ongoing research and technological developments that are steadily increasing the viability and efficiency of this approach. Biosurfactants made from recycled materials are an example of how they contribute to environmental sustainability and the circular bioeconomy. This method looks forward to developing more environmentally friendly and sustainable surfactants that have a wide range of uses in various industries. In order to ensure future prospects and sustainable growth, it is imperative to adhere to standards, such as environmental sustainability. Biosurfactants, which are expected to gain more popularity in industries aiming to reduce their environmental impact, should be embraced. It is in line with the concepts of sustainability and the circular economy. Agro-industrial waste can be fully utilized as a valuable resource for the manufacturing of biosurfactants with more research and development in this area. Biosurfactant synthesis may see a considerable drop in price if agro-industrial waste were used as a raw source. Consequently, biosurfactants may prove to be more financially feasible than their synthetic counterparts.

## Author contributions

TS: Conceptualization, Writing – review & editing, Data curation, Formal analysis, Investigation, Methodology, Writing – original draft. RG: Data curation, Investigation, Methodology, Software, Writing- original draft. SV: Data curation, Formal analysis, Methodology, Writing – original draft. VK: Formal analysis, Investigation, Methodology, Writing – original draft. SN: Data curation, Investigation, Methodology, Validation, Writing – review & editing. GG: Data curation, Methodology, Validation, Writing – review & editing. K-HB: Conceptualization, Formal analysis, Project administration, Supervision, Validation, Writing – review & editing. SR: Conceptualization, Writing – review & editing, Funding acquisition, Resources, Supervision.
